# Combination of daratumumab, ixazomib, and lenalidomide with or without dexamethasone for initial therapy of newly diagnosed myeloma

**DOI:** 10.1038/s41408-026-01567-y

**Published:** 2026-07-18

**Authors:** Shaji Kumar, Betsy Knopf, Erik Asmus, Eli Muchtar, Francis K. Buadi, Wilson Gonsalves, Melanie Thompson, David Dingli, Ronald Go, Rahma Warsame, Taxiarchis Kourelis, Lisa Hwa, Amie Fonder, Miriam Hobbs, Angela Dispenzieri, Suzanne Hayman, Nelson Leung, Moritz Binder, S. Vincent Rajkumar, Morie Gertz, Prashant Kapoor

**Affiliations:** 1 Division of Hematology, Mayo Clinic, Rochester, MN USA; 2 Department of Biostatistics, Mayo Clinic, Rochester, MN USA

**Keywords:** Chemotherapy, Myeloma

## Abstract

Quadruplets incorporating anti-CD38 monoclonal antibody have led to improved outcomes, potentially allowing limited-duration therapy and reduction in steroid doses. We designed this trial to examine the efficacy of the quadruplet regimen containing daratumumab, ixazomib, lenalidomide, and dexamethasone (Dara-IRd) given for fixed duration and to evaluate early discontinuation of steroids. Patients with untreated MM were enrolled, irrespective of their transplant eligibility. The primary objective was to determine the ≥complete response (CR) rate. Treatment involved 28-day cycles of ixazomib 4 mg days 1, 8, 15; lenalidomide 25 mg days 1–21, dexamethasone 40 mg, weekly and daratumumab 16 mg/kg, weekly for two cycles, every other week during cycles 3–6 and every 4-weeks thereafter during induction (12-cycles) followed by daratumumab and ixazomib maintenance (24-cycles). Seventy-eight patients were enrolled into two sequential cohorts: cohort A (*n* = 38) and B (*N* = 40), with dexamethasone given only for two cycles in Cohort B. At the time of the data cut-off, all patients had completed the treatment. The overall response rate was 96%, including a ≥CR rate of 32%, similar in both cohorts. Responses deepened over time; 32% achieved a marrow MRD negative status and 29% MRD_neg_-CR. After a median follow-up of 37.9 months, the median PFS or OS has not been reached. A grade ≥3 adverse event, at least possibly attributed to the study drugs, was seen in 55% of patients. The most common toxicities included fatigue, neutropenia, lymphopenia, peripheral neuropathy, diarrhea, and nausea. Stem cells were collected in 55 patients; the median sCD34+ cell yield (range) was 7.8 (2.8–15.9) × 10^6^/kg. Dara-IRd is an active regimen in newly diagnosed MM, with high overall response rate as well as deep responses. Nearly a third of the patients attained MRD_neg_ status, which improved over time. The PFS with finite-duration therapy is comparable to other studies in NDMM. Early discontinuation of dexamethasone did not impact efficacy.

## Introduction

The treatment approaches for newly diagnosed multiple myeloma (MM) have evolved over the past two decades, not just in terms of the drug classes employed but also in the use of consolidation approaches such as autologous stem cell transplantation (ASCT) in patients eligible to undergo the procedure, use of maintenance therapy after ASCT, and the duration of treatment [1]. The introduction of lenalidomide, an immunomodulatory drug (IMiD), and bortezomib, a proteasome inhibitor (PI), led to these drugs being used as doublets in combination with dexamethasone (Rd or Vd) and subsequently in triplet combination that included both drugs with steroids (VRd) [2–6]. Phase 3 trials subsequently confirmed the benefit of using a triplet over a doublet for the initial therapy of MM with improved survival [7–9]. More recently, monoclonal antibodies targeting CD38 on the myeloma cell has been added to the VRd regimen, resulting in a quadruplet regimen (DRVd) [10–12]. DRVd has been studied as initial therapy for MM prior to ASCT in phase 2 and 3 trials, demonstrating an improvement in stringent complete response and MRD negativity rates as well as progression free survival (PFS) rates compared with VRd. In addition, variations of this quadruplet using an alternate CD38 antibody (Isatuximab) or alternate proteasome inhibitor (carfilzomib) or IMiD (thalidomide) have been studied as induction therapy, all demonstrating high rates of deep response [13–19]. The standard approach for these trials, which has in turn driven the clinical practice, has been to use 4–6 cycles of induction therapy followed by a single ASCT and two cycles of post-ASCT consolidation with the same quadruplet, with de-escalation to one or two drug maintenance, either for limited duration or more often, until disease progression. Importantly, recent clinical trials have also demonstrated the value of quadruplets in patients not considered eligible for ASCT, with Daratumumab-VRd and Isatuximab-VRd improving PFS compared with VRd [12, 20].

These clinical trials have ushered in a new era of myeloma therapy, highlighted by use of quadruplet regimens in the context of ASCT, leading to deep responses and prolonged disease control, hitherto unseen among patients with NDMM [11, 15, 17, 18]. However, many questions remain unanswered. Two recent phase 3 trials using triplet induction regimens with or without ASCT have demonstrated equivalent overall survival highlighting the ability to use ASCT either as part of initial therapy or as treatment of second or subsequent relapse [8, 9]. This paradigm has not been explored in the context of the highly effective quadruplets, which on their own are capable of inducing responses as deep as those previously observed post-ASCT. The demonstration of survival benefits with the use of lenalidomide maintenance in the setting of triplet induction and ASCT led to the adoption of continuous treatment until disease progression as standard practice [21, 22]. It remains unclear if the deep responses afforded by the use of the quadruplet regimens will allow us to discontinue treatment, either after a certain period or after achieving a specific depth of response but are being explored to some extent in the ongoing randomized trials. Finally, myeloma regimens have universally included steroids, primarily dexamethasone, as part of the regimen. Dexamethasone is an effective myeloma agent and had been used as a single agent prior to the introduction of newer therapies for the initial management of myeloma [23]. Given the approach taken by myeloma investigators to build on the dexamethasone containing doublets and triplets for regulatory trials, it has continued to be a part of the current regimens. In light of the historical role that dexamethasone-containing regimens have played in securing regulatory approvals, dexamethasone has remained fundamental to the development of new treatments. But its role has become less clear as newer, more effective therapies emerge.

To develop initial data addressing these questions, we designed a 2-part phase 2 trial of ixazomib, lenalidomide, and daratumumab with or without dexamethasone. In this trial, we explored the combination of a PI and anti-CD38 monoclonal antibody as maintenance to provide complementary data to the lenalidomide combinations being explored in phase 3 trials. We elected to use ixazomib as the PI based on the data demonstrating its efficacy in combination with lenalidomide and dexamethasone in both the relapsed as well as newly diagnosed disease settings [24–27].

## Patients and methods

### Study design

This open-label, phase 2 study with two sequential cohorts evaluated the safety, tolerability, and efficacy of the combination of ixazomib, lenalidomide, daratumumab, and dexamethasone (IRd-Dara) in patients with newly diagnosed, active MM, diagnosed according to the 2014 IMWG diagnostic criteria. Patients with previously untreated MM who had measurable disease and adequate organ function were enrolled, irrespective of their eligibility for ASCT. This study accrued patients between April 2017 to March 8, 2018 (Cohort A) and June 2018 to June 2019 (Cohort B). The study was performed in accordance with the provisions of the Declaration of Helsinki, the International Conference on Harmonization, and the Guidelines for Good Clinical Practice, and with the approval of the Mayo Clinic Institutional Review Board. The study was registered at www.clinicaltrials.gov as NCT03012880.

### Study objectives

The primary objective of the study was to determine the ≥complete response (CR) rate of IRd-Dara in patients with previously untreated symptomatic MM. The secondary objectives included (1) determining the overall response rate (ORR) and very good partial response (VGPR) rate, (2) determining the progression free survival (PFS) and overall survival (OS), and (3) determining the toxicities associated with IRd-Dara in this patient population.

### Patient selection

The study enrolled patients 18 years of age or older, with symptomatic MM, who were either untreated or had received no more than one cycle of any treatment regimen. Patients were required to have measurable disease (serum M-protein ≥1 g/dL or urine M-protein ≥200 mg/24 h or involved free light chain level ≥10 mg/dL provided the serum free light chain ratio was abnormal), Eastern Cooperative Oncology Group performance status of 0–2, adequate hematologic (absolute neutrophil count ≥1000/mm^3^, platelets ≥75,000/mm^3^), hepatic (total bilirubin ≤1.5× upper limit of normal [ULN], alanine/aspartate aminotransferase ≤3× ULN), and renal (creatinine clearance ≥30 mL/min) function. Patients with grade ≥3 peripheral neuropathy or grade 2 with pain, grade >1 diarrhea, or who had major surgery or serious infection within 14 days prior to the start of therapy were excluded. Patients receiving systemic treatment with strong CYP1A2 inhibitors or strong inhibitors/inducers of CYP3A within 14 days were excluded. Other factors that precluded participation in the trial included uncontrolled cardiovascular conditions (including uncontrolled hypertension, uncontrolled cardiac arrhythmias, symptomatic congestive heart failure, unstable angina or myocardial infarction within the past 6 months), known human immunodeficiency virus positivity, known hepatitis B surface antigen-positive status, or known or suspected active hepatitis C infection, and known allergy to any of the study medications, their analogues, or excipients in the various formulations. Other comorbidities or severe pre-existing illnesses that, in the treating physician’s opinion, could interfere with oral absorption and/or tolerance of ixazomib citrate or lenalidomide excluded patients from participation.

### Drug administration

Treatment involved 28-day cycles consisting of ixazomib 4 mg days 1, 8, 15; lenalidomide 25 mg days 1–21, dexamethasone 40 mg, weekly and daratumumab 16 mg/kg, weekly for two cycles, every other week during cycles 3–6 and then every 4 weeks thereafter during induction (12-cycles) followed by daratumumab and ixazomib maintenance (maximum 24 cycles). All patients went off treatment at the completion of 36 cycles (~3 years). In cohort B, dexamethasone was discontinued after two cycles. Lenalidomide was dose-reduced to 10 mg for creatinine clearance 30–59 ml/min. Patients were allowed to collect stem cells for cryopreservation after 4 cycles if considered eligible for ASCT. Therapy could be interrupted for up to 4 weeks for the purpose of stem cell collection. Patients went off treatment to event monitoring if at least a MR (minimal response) was not seen after 2 cycles and a PR was not seen after 4 cycles.

Standard infusion reaction prophylaxis was instituted prior to daratumumab infusions. Standard anti-emetics, including 5-hydroxytryptamine 3 serotonin receptor antagonists, were recommended for emesis if it occurred once treatment was initiated; prophylactic anti-emetics could also be considered at the physician’s discretion. Prophylactic antidiarrheals were not used; however, administering antidiarrheals was allowed after infectious causes were excluded. Topical steroids and other symptomatic measures were permitted to manage any skin rash. Prophylaxis with acyclovir was used while on study therapy and for 3 months beyond the end of therapy.

### Assessments

Adverse events (AEs) were graded using the National Cancer Institute’s Common Terminology Criteria for AEs, version 4.0. Myeloma disease response was done in accordance with the International Myeloma Working Group uniform criteria. All response categories required confirmation of the required tests, except for the bone marrow used for CR determination. At any point in treatment, patients suspected of experiencing PD had response assessments repeated to confirm disease progression, done at least 1 week apart. Unlike prior studies, serum immunofixation was assessed with mass spectrometry (MASS-FIX) and marrow MRD negativity by Euro flow (sensitivity of 1 in 10^5^). Bone marrows were performed at the end of 4, 12 cycles, after 12 and 24 cycles of maintenance and only required to document CR after that. MRD testing was performed on all bone marrows, but only those in CR were considered MRD negative consistent with IMWG definition.

### Statistical analyses

The primary endpoint of this study was the confirmed ≥CR rate, where. a confirmed CR is defined as the objective status of CR or sCR that is maintained on two consecutive evaluations. All patients meeting the eligibility criteria, who have signed a consent form and have begun treatment, were evaluated for response unless there was a major violation. The sample size for the study was calculated using a one-stage binomial design and was evaluated in each cohort independently. With 36 evaluable patients, the study provided 89% power to test the null hypothesis that the CR rate is at most 25% versus the alternative hypothesis that the CR rate is at least 45%, with a 1-sided significance level of α = 0.09. An additional 4 patients were enrolled in each cohort to account for canceled or ineligible patients and protocol violations, for a total of 80 patients overall. All eligible patients who received at least 1 dose of the study drug(s) were included in the analysis for toxicity assessment. Overall survival (OS) was defined as the time from study entry to death due to any cause. Progression free survival (PFS) was defined as the time from study entry to disease progression or death due to any cause.

## Results

### Patients

Overall, 80 patients were enrolled, 40 in each cohort. One patient in cohort A was considered ineligible, and one patient withdrew consent and was not treated; they were excluded from all analyses, leaving 38 patients in Cohort A. Table 1 describes the baseline demographic and disease characteristics at study entry as well as disposition data. At the time of the data cutoff, all patients had completed the active treatment phase (36 cycles maximum), and all patients have completed follow up. The reasons for discontinuation among patients in Cohorts A and B, respectively, were disease progression in 9 and 10 patients and alternate therapy (most often transplant) in 11 and 8 patients.

**Table 1:** Baseline characteristics.

	All patients (*n* = 78)	Cohort A (*N* = 38)	Cohort B (*N* = 40)
Age (Median, range), years	63.5 (33, 81)	62.0 (41, 81)	64.5 (33, 81)
Male *n* (%)	43 (55.1%)	18 (47.4%)	25 (62.5%)
ECOG performance status (*n*) (%)			
0	38 (48.7%)	14 (36.8%)	24 (60.0%)
1	34 (43.6%)	19 (50.0%)	15 (37.5%)
2	6 (7.7%)	5 (13.2%)	1 (2.5%)
mSMART High Risk, *n* (%)^a^	26 (33.3%)	9 (23.7%)	17 (42.5%)
R-ISS *n* (%)			
1	23 (29.5%)	13 (34.2%)	10 (25.0%)
2	49 (62.8%)	20 (52.6%)	29 (72.5%)
3	6 (7.7%)	5 (13.2%)	1 (2.5%)
Serum LDH, (median, range) U/L	147 (74, 633)	158 (97, 633)	145 (74, 241)
Serum creatinine (median, range) mg/dL	1.0 (0.6, 1.6)	1.0 (0.6, 1.4)	1.0 (0.6, 1.6)
Parameter for response, *n* (%)			
Serum M-spike ≥ 1 g/dL and urine M-spike ≥ 200 mg/24 h	18 (23.1%)	9 (23.7%)	9 (22.5%)
Serum M-spike ≥ 1 g/dL only	48 (61.5%)	23 (60.5%)	25 (62.5%)
Urine M-spike ≥ 200 mg/24 h only	9 (11.5%)	4 (10.5%)	5 (12.5%)
Serum free light chain ≥ 10 mg/dL	3 (4%)	2 (5.3%)	1 (2.5%)
No disease progression (%)	59 (75.6%)	29 (76.3%)	30 (75.0%)
Alive *n* (%)	73 (93.6%)	34 (89.5%)	39 (97.5%)
Months of follow-up (alive patients) median (range), months	37.9 (36.1, 42.5)	37.8 (36.1, 42.5)	38.0 (36.2, 41.9)
Treatment cycles, median (range)	28.5 (4, 36)	24 (4, 36)	34 (4, 36)

^a^Presence of t(4;14), t(14;16), 1q gain/amplification, Del 17p.

### Response to therapy and survival

**Fig. 1:**
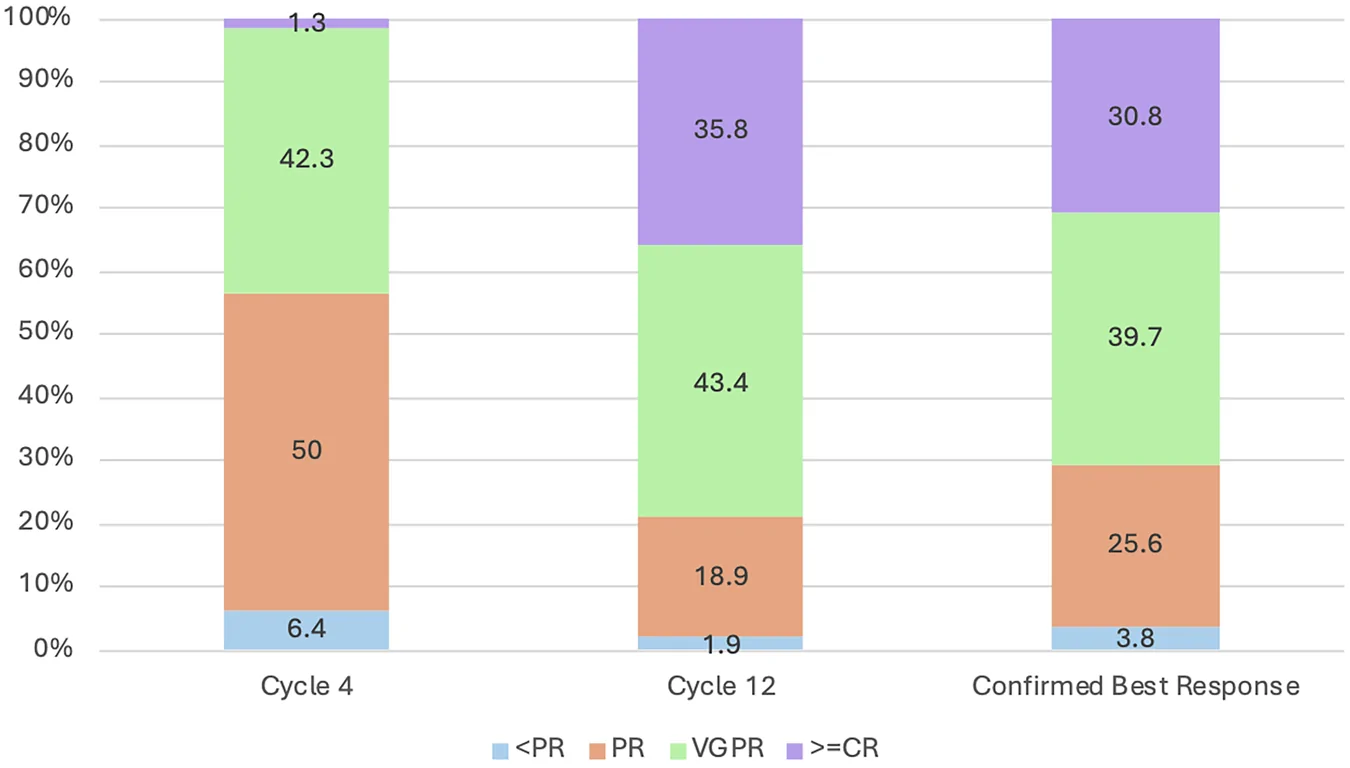
Response to treatment. Response rates, after cycle 4, 12 and the best response rate during the trial; across the two cohorts.

**Fig. 2:**
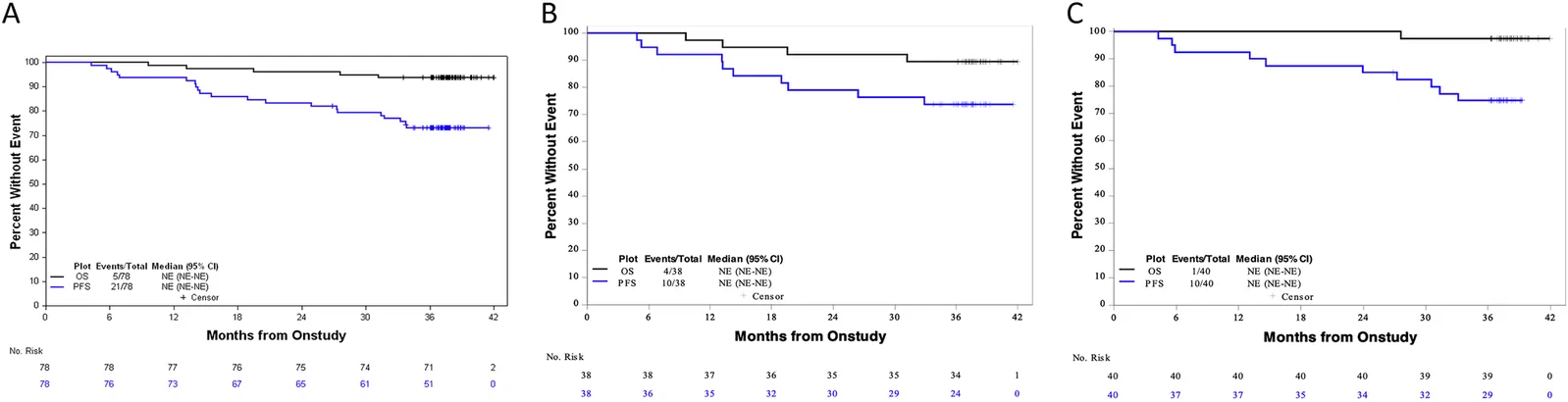
Survival Outcomes. Survival curves, both PFS and OS, for the entire trial population (Panel **A**), cohort A (Panel **B**) and Cohort B (Panel **C**).

**Fig. 3:**
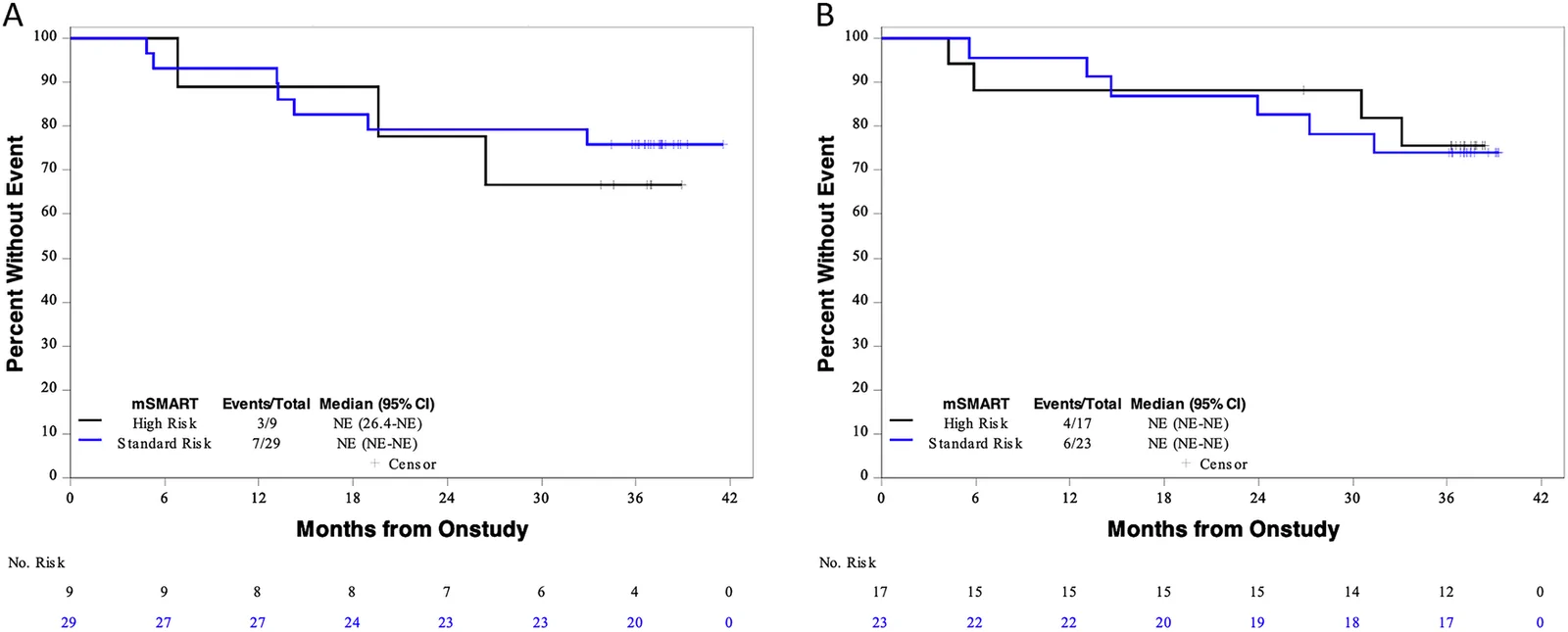
Survival outcomes by risk. Survival curves by mSMART risk category, by cohort A (Panel **A**) and Cohort B (Panel **B**).

Overall, 37 (97%) and 38 (95%) patients from Cohort A and B, (combined 96.6%) achieved a partial response or better across the entire trial, including 32% and 30% with ≥CR in Cohorts A and B, respectively (Table 2, Fig. 1, supplementary figure 1). For both cohorts, the time to response was fast; the median time to ≥CR was 8.5 cycles. Responses deepened over the duration of treatment as shown in Fig. 1; 32% of patients achieved a marrow MRD negative status (29% CR by mass spec and marrow MRD_neg_). No impact of cytogenetic risk or age on the response rate was observed. At a median follow-up of 37.9 months, the median PFS or OS are yet to be reached (Fig. 2A–C). The progression free survival rate at 3 years, with all patients off treatment, was 74% and 75%, respectively, for Cohorts A and B. No impact of the cytogenetic risk was observed in either Cohort on PFS or OS (Fig. 3A, B). We also examined the impact of depth of response on PFS using a 1-year landmark analysis. Patients in a CR at 1 year had a significantly improved PFS at 2 and 3 years compared to the those with <CR, *p* = 0.02 (Supplementary Fig. 2)

**Table 2:** Response to therapy.

Overall best confirmed response	Cohort A (*n* = 38)	Cohort B (*n* = 40)
sCR	10	8
CR	2	4
VGPR	16	15
PR	9	11
SD		1
MR	1	1
CR+ response rate, 95% CI	12 (32%)	12 (30%)
VGPR+ response rate, 95% CI	28 (74%)	27 (68%)
PR+ response rate, 95% CI	37 (97%)	38 (95%)
MRD− rate, 95% CI	11 (29%)	14 (35%)

### Dose intensity

Dose reductions were not frequent, and majority of the patients were able to maintain the dose intensity across the trials. The distribution of patients with dose modification are as shown in Table 3.

**Table 3:** Dose intensity.

A Cohort A (*n* = 38)				
	Ixazomib	Lenalidomide	Daratumumab	Dexamethasone
No. of cycles administered	836	383	869	497
Median dose	12	525	1363	160
Minimum	0	0	0	0
Maximum	12	525	9600	160
No. of patients with adjustments	7 (18%)	13 (34%)	1 (3%)	15 (39%)
Total reductions	11	17	1	19

B Cohort B (*n* = 40)				
	Ixazomib	Lenalidomide	Daratumumab	Dexamethasone
No. of cycles administered	979	405	979	637
Median dose	12	525	1488	40
Minimum	0	0	0	4
Maximum	12	550	10,568	160
No. of patients with adjustments	8 (20%)	8 (20%)	(%)	11 (28%)
Total reductions	9	10		15

### Adverse events

**Fig. 4:**
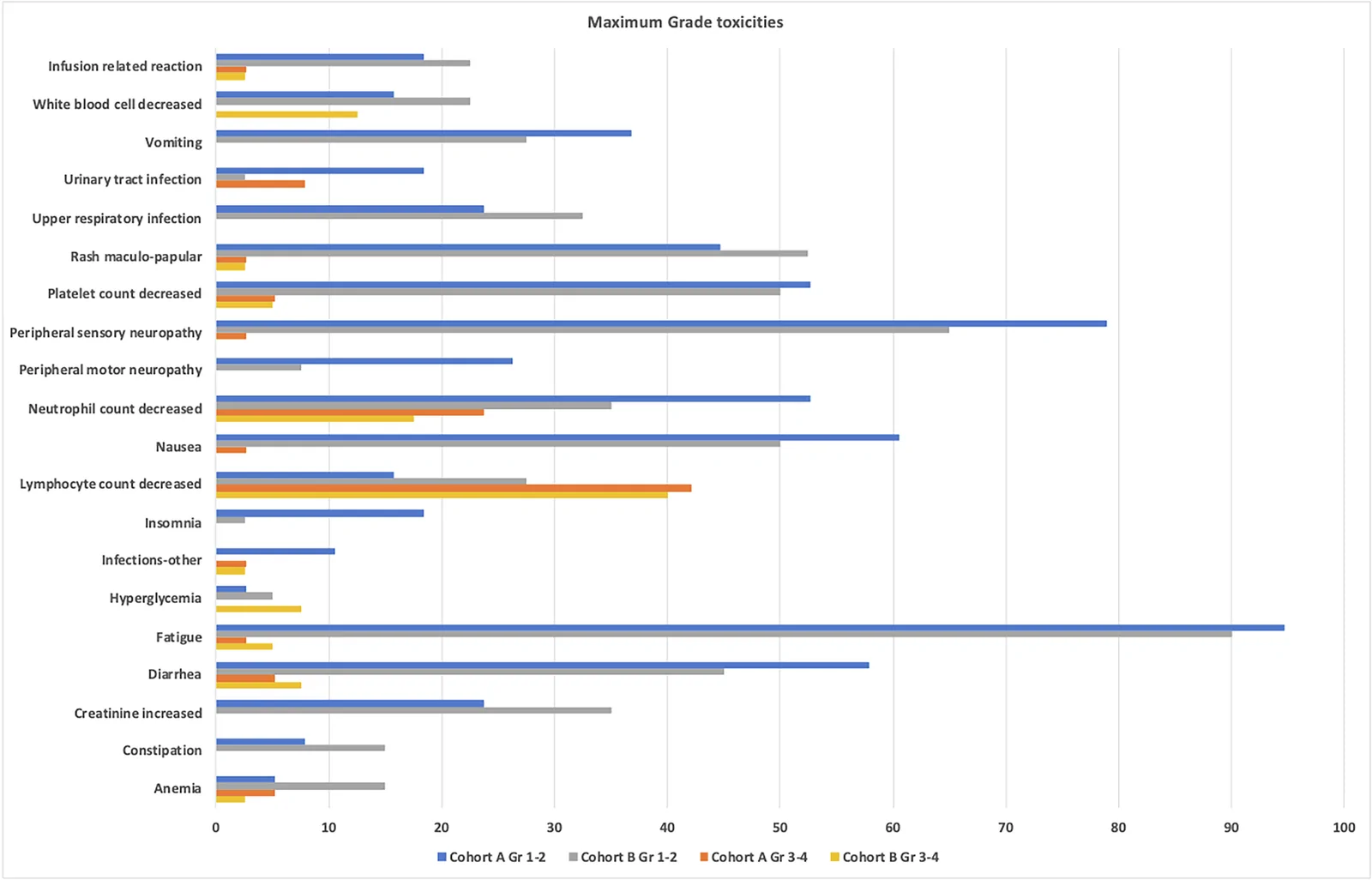
Adverse Events. Toxicity profile of treatment across the two cohorts.

A grade 3 or higher adverse event at least possibly attributed to the study drugs was seen in 55% of patients. Table 4 provides the distribution of all grades of toxicities considered at least possibly related to the drug administration. The most common toxicities included fatigue, neutropenia, lymphopenia, peripheral neuropathy, diarrhea, and nausea (Fig. 4).

**Table 4:** Adverse events across the study.

	Regardless of attribution		At least possibly related	
	Cohort A	Cohort B	Cohort A	Cohort B
Grade 3+ adverse events	26 (68%)	23 (58%)	22 (58%)	21 (53%)
Grade 4+ adverse events	7 (18%)	5 (13%)	5 (13%)	5 (13%)
Grade 5 adverse events	0 (0%)	0 (0%)	0 (0%)	0 (0%)
Grade 3+ heme AE	21 (55%)	18 (45%)	18 (47%)	18 (45%)
Grade 4+ heme AE	5 (13%)	5 (13%)	5 (13%)	5 (13%)
Grade 5 heme AE	0 (0%)	0 (0%)	0 (0%)	0 (0%)
Grade 3+ non-heme AE	16 (42%)	11 (28%)	10 (26%)	8 (20%)
Grade 4+ non-heme AE	5 (13%)	0 (0%)	1 (3%)	0 (0%)
Grade 5 non-heme AE AE	0 (0%)	0 (0%)	0 (0%)	0 (0%)

Stem cell collection

Stem cells were collected in 55 patients; median (range) CD34+ yield was 7.8 (2.8, 15.9) ×10^6^/kg with plerixafor used in 87% of patients as per our standard of care “on-demand” approach based on peripheral blood CD34+ cell count post G-CSF [28].

## Discussion

The current study explored a quadruplet regimen in an all-comer population, regardless of their eligibility for ASCT, while allowing patients who are considered transplant-eligible to collect stem cells early and store them for future use. With 2 large phase 3 trials demonstrating no overall survival detriment to delaying ASCT, there is an increasing trend toward collecting and storing for future transplant, and this study provides valuable information for such an approach [8, 9]. Several phase 2 and 3 trials have explored the use of quadruplet induction therapy prior to planned ASCT, but the use of a quadruplet regimen in the context of delayed ASCT has yet to be explored to the same extent [10, 11]. The trial is unique in that it explored the use of a quadruplet without planned ASCT, defined duration of treatment, early discontinuation of steroids in the quadruplet setting, and finally examined the feasibility and efficacy of an oral proteasome inhibitor in combination with daratumumab as maintenance therapy in contrast to lenalidomide alone or in combination with daratumumab. The use of the oral proteasome inhibitor allowed patients to receive only a monthly injection of daratumumab, with an otherwise all-oral regimen.

The trial results provide important information for the current practice. The regimen demonstrated efficacy with nearly all patients achieving a partial response or better however as compared to the more recent quadruplet therapy read-outs, the current study’s CR rates appear to be lower than seen with phase 3 trials like PERSEUS, but similar to the AFT-41 trial using the same combination [29]. This at least partly reflects the use of mass spectrometry for detecting monoclonal protein, a test with at least 1 log more sensitivity than traditional immunofixation testing. Looking at the VGPR rate, ~70% of patients achieved VGPR. The lack of incorporation of ASCT also contributes to a reduced depth of response, as high-dose chemotherapy deepens the response, as is evident from the randomized trial of ASCT versus delayed ASCT/none. Nearly a third of the patients were MRD negative in the bone marrow and had no detectable monoclonal protein by mass spectrometry. Furthermore, the progression-free survival rates at the end of the planned treatments (36 cycles) demonstrate that nearly 80% of the patients are free from progression, with over 90% of patients alive at that time.

An important question for the field is the ideal duration of treatment in patients with newly diagnosed MM. While the results are not definitive, the current results provide an estimate that can provide critical information for future trial designs. In the PERSEUS trial, patients who are MRD negative at the end of 3 years of treatment could discontinue daratumumab, though the impact of this is unclear with the current follow-up and the available results [11]. In the MASTER trial, patients receiving a quadruplet induction and transplant could discontinue therapy if MRD is negative over a 4-month period [17]. While MRD-guided approaches will be critical for future practice, definitive answers are unavailable. Here, we explored a defined duration of 36 cycles with all patients going off treatment at that time.

Even though the trial was not designed to compare the efficacy and toxicity between the two cohorts formally, the discontinuation of weekly dexamethasone after cycle 2, except for a small dose used as infusion reaction prophylaxis, did not impact the response rate or depth or the risk of progression and led to a decrease in the grade 3+ event rate. Increasingly, trials have demonstrated that more modern treatments can allow us to reduce the overall use of steroids. The ECOG E4A03 trial demonstrates the superiority of weekly dexamethasone over the more intense schedule in terms of survival despite deeper response [2]. More recently, a phase 3 trial demonstrated discontinuation of steroids after 9 cycles was associated with better event-free survival [30]. As the efficacy of the modern combinations increases, the incremental value of steroids appears to be shrinking, and early discontinuation can potentially reduce toxicity, especially immunosuppression and bone loss with long-term steroids.

The role of a PI during maintenance remains rather undefined. In the retrospective series, the use of a PI with lenalidomide appears to be beneficial for those with high-risk disease when compared with standard-risk patients [31]. Given the good outcomes seen with daratumumab and lenalidomide in MAIA trial among high-risk patients, it remains unclear if this is the impact of the PI or just the reflection of the use of 2 different drugs with different mechanisms of action allowing for better control of the tumor clone. In the PETHEMA trial, the use of ixazomib with lenalidomide and dexamethasone did not appear to improve outcomes compared with lenalidomide alone [32]. However, that trial also used dexamethasone, which is typically not used with the current maintenance regimens, making that data less generalizable. While a comparison of efficacy is not possible between lenalidomide used in combination with daratumumab, the current trial demonstrates the feasibility of daratumumab-ixazomib combination.

In summary, we demonstrate activity of a quadruplet using an oral PI in a population of newly diagnosed MM irrespective of transplant eligibility, with a fixed duration treatment and delayed ASCT for those considered eligible. In addition, we provide data in support of early discontinuation of dexamethasone when used in combination with 3 active drugs classes, without loss of efficacy.

## Supplementary information

Supplementary figures

## Data Availability

Trial-level data are available on request
