# Supplementary material for: Combination of daratumumab, ixazomib, and lenalidomide with or without dexamethasone for initial therapy of newly diagnosed myeloma
Source: Blood Cancer J. 2026 Jul 18;16(1):180. doi: 10.1038/s41408-026-01567-y (PMC13661603; doi:10.1038/s41408-026-01567-y)
Supplement: Supplementary file 1 [file 41408_2026_1567_MOESM1_ESM.docx]

**Supplementary figures**

**Figure S1.** Panels A and B shows the waterfall plot depicting the reduction of the paraprotein during therapy.

**Supplementary Figure 1A**

**Supplementary Figure 1B**

**Supplementary Figure 2**
